# Different Members of a Simple Three-Helix Bundle Protein Family Have Very Different Folding Rate Constants and Fold by Different Mechanisms

**DOI:** 10.1016/j.jmb.2009.05.010

**Published:** 2009-07-31

**Authors:** Beth G. Wensley, Martina Gärtner, Wan Xian Choo, Sarah Batey, Jane Clarke

**Affiliations:** Department of Chemistry, MRC Centre for Protein Engineering, University of Cambridge, Lensfield Road, Cambridge CB2 1EW, UK

**Keywords:** hTRF1, human TRF1 Myb domain, En-HD, engrailed homeodomain, PSBD, peripheral subunit-binding domain, RCO, relative contact order, LRO, long-range order, PDB, Protein Data Bank, protein folding, phi-value, chevron, folding mechanism, α-helix

## Abstract

The 15th, 16th, and 17th repeats of chicken brain α-spectrin (R15, R16, and R17, respectively) are very similar in terms of structure and stability. However, R15 folds and unfolds 3 orders of magnitude faster than R16 and R17. This is unexpected. The rate-limiting transition state for R15 folding is investigated using protein engineering methods (Φ-value analysis) and compared with previously completed analyses of R16 and R17. Characterisation of many mutants suggests that all three proteins have similar complexity in the folding landscape. The early rate-limiting transition states of the three domains are similar in terms of overall structure, but there are significant differences in the patterns of Φ-values. R15 apparently folds via a nucleation–condensation mechanism, which involves concomitant folding and packing of the A- and C-helices, establishing the correct topology. R16 and R17 fold via a more framework-like mechanism, which may impede the search to find the correct packing of the helices, providing a possible explanation for the fast folding of R15.

## Introduction

Much has been learned about the folding of proteins from comparative studies of the folding of proteins that are related in structure—proteins that have the same fold.^1,2^ There are two complimentary approaches that can be considered: comparison of proteins with the same fold and comparison of different folds. These approaches combine to yield details of features that are conserved at each level and differences that are possible. Such questions may, perhaps, be easier to address for simple systems such as the three-helix bundle. One such fold that has been studied in detail is the homeodomain-like superfamily. In this family, human TRF1 Myb domain (hTRF1) folds slowly with two-state kinetics via a nucleation–condensation mechanism from a denatured state that does not contain residual structure.^3^ At the other extreme is engrailed homeodomain (En-HD), which folds fast through a framework mechanism, has observable multistate kinetics, and extensive residual helical structure.^3–8^ Between these two extremes lies the protein c-Myb-transforming protein, which sits on a continuum between the two extremes of En-HD and hTRF1, with certain features unifying the transition states of all three domains.^3,9^ An important observation is that, for these proteins, there is a correspondence between intrinsic helical propensity, folding mechanism, and folding rate constants: En-HD has a high intrinsic helical propensity, leading to a tendency to fold via a diffusion–collision framework mechanism, which results in fast folding. hTRF1 has a low helical propensity, such that individual helices are unstable in the absence of tertiary interactions, thus requiring a nucleation–condensation mechanism for folding—leading, it is proposed, to slower folding.

Similar trends have been observed for the peripheral subunit-binding domain (PSBD) family, which has two α-helices and a 3_10_-helix. Although not as extensively studied as the homeodomain family, these domains also show some evidence for a continuum of mechanism from nucleation–condensation to framework and, again, the same relationship to folding rate constants.^10–12^

**Fig. 1:**
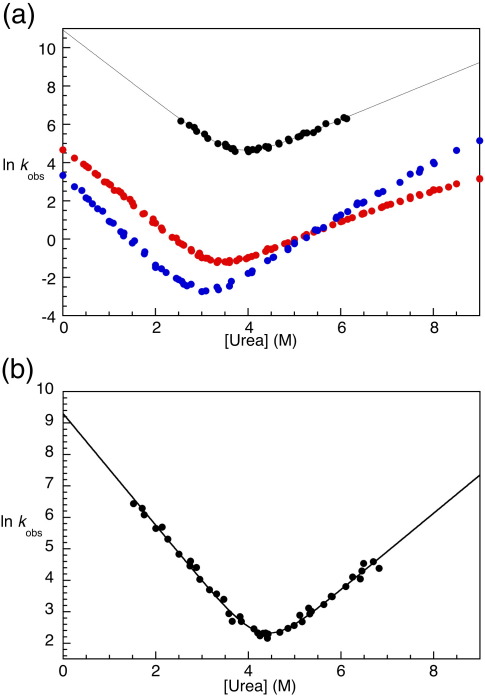
Kinetics of wild-type spectrin domains. (a) The three wild-type spectrin domains at 25 °C. R15 (black) (un)folds approximately 3 orders of magnitude faster than R16 (red) and R17 (blue). No data were collected when the rate constant exceeded 660 s^− 1^, as this is the limit of accuracy of our stopped-flow instruments. The continuous black line represents the fit of the R15 data to a standard two-state folding model, aiding the comparison of R15 with R16 and R17. (b) Wild-type R15 at 10 °C. No data are included where the rate constants are < 660 s^− 1^, and no data are collected at urea concentrations over 7 M due to potential mixing artifacts caused by viscous solutions at low temperatures. The continuous line again represents the fit of the data to a standard two-state folding model. The folding and unfolding arms of R15 that are accessible at 10 °C are longer than those at 25 °C, but are still relatively short compared with those of R16 and R17. Data for (a) were taken from Scott *et al.*^16^

Another three-helix bundle fold that has been studied extensively is the spectrin-like fold.^13–16^ Spectrin domains have about twice as many residues as those described above, and so the helices they contain are significantly longer and the domains are rod-shaped.^17–24^ Three domains—the 15th, 16th, and 17th tandem repeats from chicken brain α-spectrin (R15, R16, and R17, respectively)—have been studied in our laboratory. Although they have approximately 30% pairwise sequence identity and very similar structures (pairwise backbone RMSD < 1 Å), stabilities, and β-Tanford values, R15 folds and unfolds some 3 orders of magnitude faster than R16 and R17 (Fig. 1).^16^ This is a large difference when compared with, for instance, the homeodomain family, in which folding rate constants span just over 2 orders of magnitude.

The reason for this huge discrepancy in folding and unfolding rate constants is not immediately obvious. There are no obvious significant differences in the native state or in intrinsic helical propensity; moreover, unlike Ig-like domains, where the folding rate constants vary over about 5 orders of magnitude but are strongly correlated to stability,^25,26^ spectrin domains have similar free energies of unfolding. Since both the folding rate constant and the unfolding rate constant of R15 are significantly faster than those of R16 and R17, it seemed likely that the difference is due to differences in the folding mechanism and/or energy/structure of the transition state for folding. This is investigated in the study presented here.

A relationship between native-state topology and folding rate for small two-state proteins was established by Plaxco *et al.*^27^ They developed the concept of relative contact order (RCO) as a measure of topological complexity. A more recent analysis using a larger, more varied data set found that long-range order (LRO) gave a stronger correlation with a folding rate constant for the proteins analysed.^28,29^ LRO only considers contacts that have a sequence separation of 12 or more residues. The structures of R15, R16, and R17 are very similar; thus, the RCO and LRO for each domain are comparable. The use of each domain in isolation from the Protein Data Bank (PDB) structure 1u4q gives an RCO of around 9.5% and an LRO of around 0.4. It is interesting to note that, irrespective of whether RCO or LRO is used to quantify native-state topology, R15 falls within the scatter of the large data set used by Istomin *et al.*, whereas R16 and R17 are slow-folding outliers.^28^ The pertinent question to ask about the folding and unfolding rates is therefore: Why do R16 and R17 fold so slowly? In order to answer this, the folding of the possibly “more typical” R15 must be investigated.

Accordingly, a Φ-value analysis of R15 is presented here and compared with those of R16 and R17.^13,15^

## Results

### Wild-type R15

R15 folds relatively fast on a stopped-flow timescale, so all work in this study has been carried out at 10 °C, unless otherwise stated, to expand the range of kinetic data accessible. Equilibrium studies of R15 at 10 °C show that it folds reversibly and cooperatively as a two-state system, as has been seen at 25 °C.^16^ At 10 °C, both folding and unfolding traces fit well to single-exponential kinetics, and the resulting chevron plot appears linear within the range of data accessible (Fig. 1b). Data points with (un)folding rate constants over 660 s^− 1^ were excluded, as these points are too fast to be accurately determined by our stopped-flow instruments. Also, no data were collected for denaturant concentrations > 7 M urea due to potential inaccuracies caused by poor mixing of viscous solutions at low temperatures.

Both arms of the chevron plot of wild type and most mutants of R15 appear linear (Fig. 1). The kinetic free energy of unfolding Δ*G*_kin_^H_2_O^ is smaller than the equilibrium value Δ*G*_D–N_^H_2_O^ by ∼ 1 kcal mol^− 1^ at 10 °C (7.2 *versus* 8.2 kcal mol^− 1^, respectively). A similar discrepancy is also seen at 25 °C in the free energy of unfolding, where the kinetic value Δ*G*_kin_^H_2_O^ is 6.0 kcal mol^− 1^ compared to 6.7 kcal mol^− 1^ for the equilibrium value. It is important to note, however, that the range of denaturant concentrations that can be probed by stopped-flow kinetics is relatively small, so that even at 10 °C, both chevron arms are relatively short; thus, the error introduced by extrapolation of both folding and unfolding rate constants to 0 M denaturant is significant.

### Φ-Value analysis: 1. Choice of mutations

To allow a direct comparison of this Φ-value analysis with the published Φ-value analysis of R16, we based the positions chosen for each Φ-value on those made in R16 and with reference to the crystal structure of R15 (PDB code 1u4q).^15,18^ Two classes of mutations were made, all within the three helices: conservative deletion mutations (to Ala) in the core of the protein, which act as probes of the formation of tertiary structure (packing of the helices), and Ala-Gly scanning mutations on the surface, which probe helix formation.^30,31^ Mutations in the loop regions were not attempted because of the very small ΔΔ*G*_D–N_ values observed for loop mutations in R16.^15^ In total, 66 mutations were made at 51 different sites. The contacts deleted for the core mutations are reported in Table 1.

### Φ-Value analysis: 2. Analysis of data

The stability of all mutants was determined using equilibrium denaturation in urea by monitoring fluorescence at 360 nm. The fluorescence change seen for H58A was small at all wavelengths, so the equilibrium stability of this mutant was determined by monitoring ellipticity at 222 nm. Data for each mutant were fitted individually and fitted well to a two-state transition.^32^ The mean equilibrium *m*-value *m*_D–N_^eqb^ (1.88 ± 0.15 kcal mol^− 1^ M^− 1^) is approximately the same as that of wild type (1.82 kcal mol^− 1^ M^− 1^), and the range of *m*-values is similar to that observed in other analyses of this kind. This mean value of *m*_D–N_^eqb^ was used to determine the change in free energy on mutation ΔΔ*G*_D–N_ to reduce the error (Eq. (1)):

(1) $$\text{ΔΔ}G_{\text{D–N}}=\langle m_{\text{D–N}}^{\text{eqb}}\rangle \delta {\left[\text{urea}\right]}_{50\%}$$

where 〈*m*_D–N_^eqb^〉 is the mean *m*-value and δ[urea]_50%_ is the difference in denaturation midpoint between wild-type and mutant proteins. Note that for Ala-Gly mutant analysis, δ[urea]_50%_ is the difference between [urea]_50%_ for the Ala and Gly mutations.

The folding and unfolding kinetics of all mutants were followed by fluorescence and fitted well to single-exponential kinetics. The refolding arms of all the chevrons are linear within the range of the data and have the same *m*_*k*___f__ within error when fitted individually to a two-state model (Eq. (2)). Some chevrons (V25A, E26G, A53G, Q95G, and I97A) suggest a slight curvature in the unfolding limb; however, as the limbs are short, this curvature cannot be fitted with any accuracy. Thus, all chevron plots were fitted assuming linear folding and unfolding arms. There is a significant variation in *m*_*k*___u__, although the length of the two limbs is comparable for the majority of the mutants. Consequently, all the data (wild type and mutants) were fitted globally to the two-state model, sharing *m*_*k*___f__ to reduce the errors of the fits. The shared *m*_*k*___f__ was 1.84 ± 0.01 M^− 1^, and the *m*_*k*___u__ values were in the range of 0.72–1.46 M^− 1^. As seen for wild-type R15, the equilibrium Δ*G*_D–N_^H_2_O^ values Δ*G*_D–N_^H_2_O^ for the mutants are consistently higher than the kinetic values Δ*G*_kin_^H_2_O^. The equilibrium *m*-values *m*_D–N_^eqb^ for the set of wild type and mutants are also consistently higher than the kinetic values *m*_D–N_^kin^, where *m*_D–N_^kin ^= *RT*(*m*_*k*___f__ + *m*_*k*___u__) (Fig. S1a). This discrepancy is consistent but not large. It can only be observed above the noise due to the large number of mutants characterised.

(2) $$\text{ln}k_{\text{obs}}=\text{ln}\left(k_{f}^{H_{2}O}\text{exp}\left(-m_{k_{f}}\cdot [\text{urea}]\right)+k_{u}^{H_{2}O}\text{exp}\left(m_{k_{u}}\cdot [\text{urea}]\right)\right)$$

**Fig. 2:**
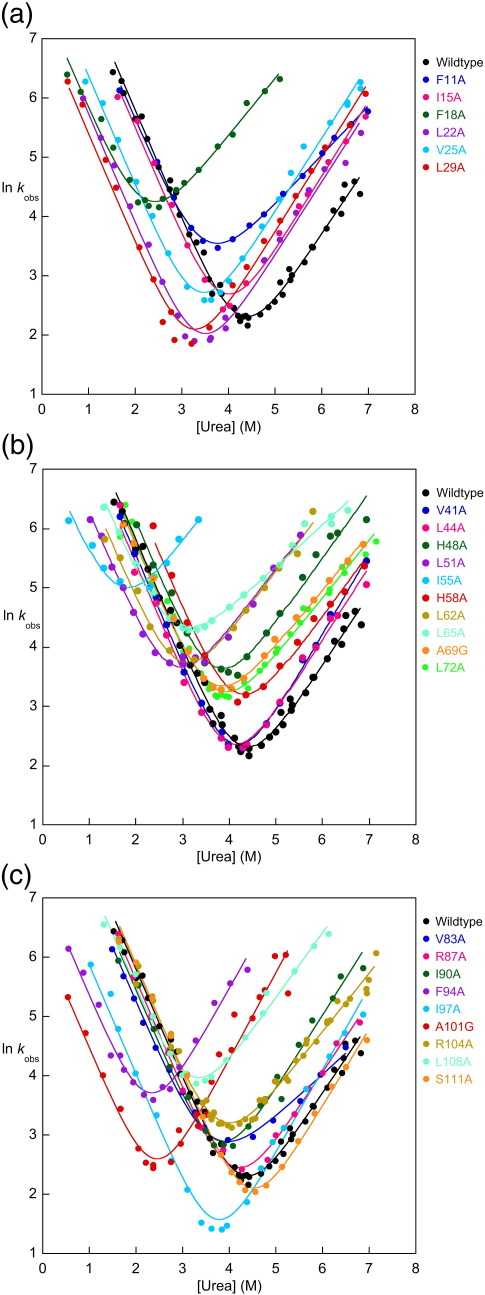
Chevron plots and fits for core mutants. (a) Core mutants in helix A; (b) core mutants in helix B; (c) core mutants in helix C. Continuous lines represent the fit for each mutant to a globally fitted two-state fit with a shared *m*_*k*___f__.

Chevron plots for all core mutations are shown in Fig. 2, and chevron plots for all Ala-Gly mutations are shown in Fig. S2. Thermodynamic and kinetic parameters derived from fits of the experimental data are shown for the core mutants in Table 2 and for the Ala-Gly scanning mutants in Table 3.

Φ-Values were determined from folding data (Eq. (3)) and from unfolding data at 6 M urea (Eq. (4)) using the ΔΔ*G*_D–N_ value determined using equilibrium denaturation. The overall magnitude and pattern of the Φ-values are the same if the ΔΔ*G*_D–N_ value determined from kinetic data is used. If ΔΔ*G*_D–N_ is low, then the error in Φ is significant. It has been suggested that Φ-values are valid, providing ΔΔ*G*_D–N_ > 0.6 kcal mol^− 1^.^33^ From an analysis of our data (see Fig. S3 and discussion in Scott *et al.*^15^), we normally use a slightly higher cutoff, and so Φ-values were not determined where ΔΔ*G*_D–N_ < 0.75 kcal mol^− 1^.

(3) $$\Phi_{f}=\frac{RT\text{ln}\left(\frac{k_{\text{f,WT}}^{{\text{H}}_{2}\text{O}}}{k_{\text{f,mut}}^{{\text{H}}_{2}O}}\right)}{\text{ΔΔ}G_{\text{D–N}}}$$

where Φ_f_ is the Φ-value calculated from folding data, and *k*_f__,WT_^H_2_O^ and *k*_f__,mut_^H_2_O^ are the folding rate constants of wild-type and mutant proteins, respectively, in 0 M denaturant:

(4) $$\Phi_{u}^{6M}=1-\frac{-RT\ln\left(\frac{k_{\text{u,WT}}^{\text{6}M}}{k_{\text{u,mut}}^{\text{6}M}}\right)}{\text{ΔΔ}G_{\text{D–N}}}$$

where Φ_u_^6M^ is the Φ-value at 6 M urea calculated from unfolding data, and *k*_u,WT_^6M^ and *k*_u,mut_^6M^ are the unfolding rate constants of wild-type and mutant proteins, respectively, in 6 M urea. The Φ-values are reported in Tables 2 and 3. For Ala-Gly helix scanning mutations, the (un)folding rate constant of the Gly mutant was compared to that of the Ala mutant at the same position.^31^

### Altering the helical propensity of R16

**Fig. 3:**
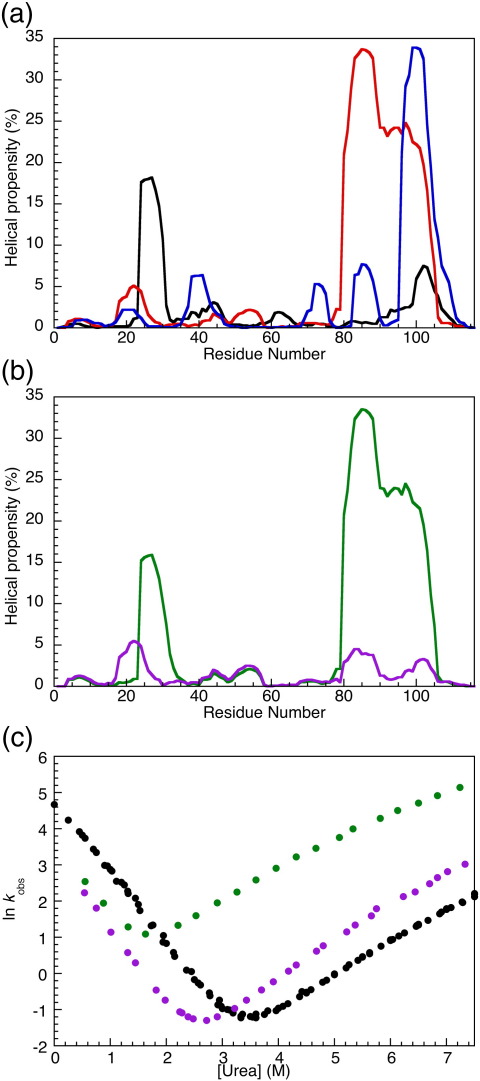
Altering the helical propensity of R16. Helical propensities calculated at 50 mM ionic strength and 25 °C using AGADIR.^34–37^ (a) Helical propensity of R15 (black), R16 (red), and R17 (blue). The mean values are 2.1% for R15, 6.3% for R16, and 3.9% for R17. (b) Helical propensities of R16rh (purple) and R16ih (green). The mean values are 1.4% for R16rh and 6.9% for R16ih. (c) Chevron plots at 25 °C for wild-type R16 (black), R16rh (purple), and R16ih (green). Alterations in helical propensity do not speed the folding of R16.

R15 differs from R16 and R17 in the distribution of helical propensity, as predicted by the program AGADIR^34–37^ (Fig. 3a). R16 and R17 show significant helical propensity in the C-helix, whereas R15 shows the greatest helical propensity in the A-helix. R16 was used to investigate whether this different distribution leads to the huge variation in (un)folding rate constants seen between R15 and R16. Two R16 variants were designed—R16rh (reduced helicity in the C-helix) and R16ih (increased helicity in the A-helix)—with altered predicted helical propensities (Fig. 3b). The helical propensity seen in the C-helix of wild-type R16 was reduced in R16rh by mutating three surface residues to glycine (Q85G, D92G, and Q99G). A more extensive redesign was required to increase the helical propensity in the A-helix of R16 to levels comparable to that seen in R15. R16ih was created by the mutation of six residues. Four surface residues were replaced with the residue seen at that position in R15 (K23S, K26E, L27A, and S30A), and one was replaced with alanine (S31A). The core mutation V29A was also made as the low helical propensity of valine prevented a significant increase in helical propensity in this region when valine was present. The single-point mutation V29A has previously been made in R16 and does not have a significant effect on the thermodynamic or kinetic properties of R16.^15^

As expected, R16rh and R16ih are less stable at equilibrium than wild-type R16 at 25 °C; Δ*G*_D–N_^R16rh ^= 4.8 ± 0.3 kcal mol^− 1^ and Δ*G*_D–N_^R16ih ^= 2.7 ± 0.1 kcal mol^− 1^ compared with Δ*G*_D–N_^WTR16^ = 6.3 ± s0.1 kcal mol^− 1^. Chevron plots of the two constructs at 25 °C both clearly display curvature in the unfolding arm (Fig. 3c), as does wild-type R16.^16^ This can be fitted using either a broad transition-state model or a sequential transition-state model with the same results.^14^ These two R16 variant chevrons were fitted using the sequential transition-state model with fixed unfolding *m*-values previously derived from the globally fitted set of R16 mutants (*m*_2_ = −0.7 M^− 1^; *m*_−_ _2_ = 0.5 M^− 1^).^15^ The folding kinetics (Fig. 3c) clearly show that neither of the alterations in AGADIR helical propensity made in these constructs has the desired effect—that of significantly increasing both the folding rate constant and the unfolding rate constant of wild-type R16. In fact, the folding rate constants in water for both R16rh and R16ih are lower than those of wild-type R16: *k*_f__,R16rh_ = 31 ± 1 s^− 1^ and *k*_f__,__R16ih_ = 42 ± 1 s^− 1^, compared with *k*_f__,WTR16_ = 125 ± 3 s^− 1^ for wild type. The unfolding rate constant in water is greater, especially for R16ih; however, this is due to destabilisation of the native state with respect to the transition state. *k*_u,R16rh_ = 8.4 × 10^− 3^ ± 0.5 × 10^− 3^ s^− 1^ and *k*_u__,R16ih_ = 0.31 ± 0.01 s^− 1^, compared with *k*_u__,WTR16_ = 3.2 × 10^− 3^ ± 0.1 × 10^− 3^ s^− 1^.

## Discussion

### Does R15 fold by a two-state mechanism?

R15 is two-state at equilibrium, and the chevron plots of both wild-type R15 and most of its mutants have linear arms, suggesting two-state kinetic behaviour. However, since both chevron arms are very short, even at 10 °C, this is not conclusive evidence that R15 shows two-state kinetic behaviour. In the first place, no refolding data could be collected at low denaturant concentrations, so the presence of a marginally stable refolding intermediate, populated at low concentrations of denaturant only, cannot be excluded. There are indications that the folding landscape of R15 is more complex than that of an ideal two-state folder. Complexity is not unexpected when R15 is considered in the context of the folding of its two homologues, R16 and R17. As both fold so much more slowly than R15, much longer chevron arms are accessible. In R16 and its mutants (and indeed in many mutants of R17), curvature in the unfolding arm, which has been interpreted in terms of either a broad transition-state model or a sequential transition-state model, can clearly be seen (e.g., Fig. 1).^13–16^

Although *m*_*k*___f__ is constant, there is considerable variation seen in *m_k_*__u__ across wild type and the set of R15 mutants. An important observation is that for a few mutants of R15, we see indications of the beginning of curvature in the unfolding arm, as is seen for R16 and for many mutants of R17.^13,15^ The most obvious explanation for these observations is that the unfolding limb for R15 does indeed display curvature, but that the unfolding limb is simply not long enough in this fast-folding protein for curvature to be observed. To examine this possibility, we refitted the data for the chevrons of wild-type R16 and its mutants, excluding data collected above 6 M urea. Now the chevrons almost all appear linear (Fig. S1d) but with *m*_*k*___u__ values that vary widely, suggesting indeed that had we been able to collect accurate unfolding data for R15 at higher urea concentrations, we might have observed a definitive curvature in the unfolding limb.

Moreover, the *m*_*k*___u__ variation in R15 is strongly correlated with the observed discrepancy between the equilibrium and the kinetic free energies of unfolding Δ*G*_D–N_^H_2_O ^− Δ*G*_kin_^H_2_O^ (Fig. S1e). Those mutants with a high *m*_*k*___u__ value have Δ*G*_D–N_^H_2_O^ ≈ Δ*G*_kin_^H_2_O^. This suggests that both folding and unfolding reflect a transition across the same free energy barrier for these variants. However, proteins with a low *m*_*k*___u__ value have a Δ*G*_kin_^H_2_O^ value that is significantly lower than Δ*G*_D–N_^H_2_O^, suggesting that the observed unfolding process monitors unfolding over a later transition-state barrier, and that the smaller values of Δ*G*_kin_^H_2_O^ are underestimations of Δ*G*_D–N_^H_2_O^ in water. This is consistent with the lower kinetic *m*-values *m*_D–N_^kin^ seen for the majority of mutants when compared with the equilibrium value *m*_D–N_^eqb^ (Fig. S1a). Similarly, when R16 and its mutants are fitted with short linear unfolding limbs, *m*_D–N_^kin^ consistently underestimates *m*_D–N_^eqb^ (Fig. S1b). When the entire length of the R16 unfolding arms is fitted with a sequential transition-state model, *m*_D–N_^kin^ is in agreement with *m*_D–N_^eqb^ (Fig. S1c).^14^

Finally, the Φ-values of R15, when calculated from folding and unfolding data, are not the same. For approximately two-thirds of the Φ-values calculated for R15, the Φ-values calculated at high denaturant using unfolding data Φ_u_^6M^ are greater than the Φ-values for folding, determined from rate constants for folding extrapolated to 0 M denaturant Φ_f_ (Tables 2 and 3). We infer that the structure present in R15 grows as it crosses the transition state, which again is evidence for a more complicated landscape than a simple two-state kinetic mechanism with a narrow transition-state barrier. Thus, the data suggest that R15 folds along a similar pathway to R16 and R17, which can be characterised either with a broad transition state or with two sequential transition states.^13–15^

### The structure of the early transition state in R15

**Fig. 4:**
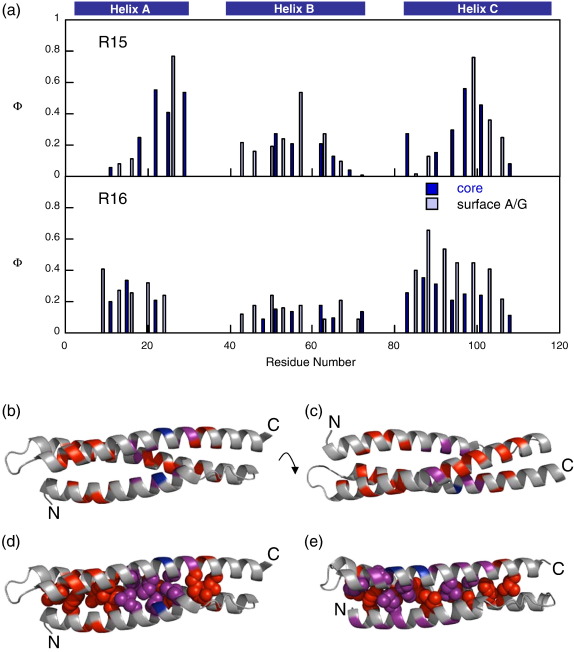
Comparison of the Φ-values of R15 and R16. (a) Histograms of Φ-values of the rate-limiting transition state at low denaturant concentrations for R15 (Φ_f_; top) and R16 (Φ_early_; bottom^15^). Core mutants are shown in dark blue, and exposed Ala-Gly mutations are shown in pale blue. (b–d) Ribbon diagrams showing the R15 Φ_f_-values mapped onto the structure of R15 (from PDB file 1u4q^18^). (b) The A-helix–C-helix interface and (c) the B-helix. (d) The same structure as in (b) showing core mutations as space-filling models. (e) Ribbon diagram showing the R16 Φ_early_-values mapped onto the R16 structure (from PDB file 1u4q), showing the A-helix–C-helix interface and core mutations presented as space-filling models. Low Φ-values (0.0–0.3) are shown in red, medium Φ-values (0.3–0.6) are shown in purple, and high Φ-values (0.6–1.0) are shown in blue.

Although folding and unfolding Φ-values have been determined for R15, only the Φ_f_-values can be analysed in detail in a similar way to the experimental Φ-value analysis of R17.^13^ Irrespective of the precise nature of the energy barrier to the (un)folding of R15, these Φ_f_-values report on the rate-limiting transition state of R15 at low denaturant concentrations (note that *m*_*k*___f__ values are the same for all mutant proteins characterised, suggesting that they all fold via the same transition state at low denaturant concentrations). Ala-Gly scanning of surface helical residues has been used extensively to probe the formation of secondary structure in the transition state.^30,31,38,39^ The truncation of buried residues to Ala reports on the packing of the helices in the transition state. There is no difference between the pattern of Φ_f_-values seen from core mutations and the pattern of Φ_f_-values seen from Ala-Gly scanning mutations in any of the helices, suggesting that secondary and tertiary structure form concomitantly (Fig. 4). There are two regions of high Φ-values: at the C-terminal end of the A-helix centred round E26A/G and in the centre of the C-helix centred on S99A/G. These two regions of structure are close together in the native state of R15, so we infer that they interact in a native-like way at the transition state (Fig. 4b and d). Tertiary contacts between helices A and C are formed and, concomitantly, approximately three turns of each helix form. The Φ-values decrease away from these two centres so that low Φ-values are seen in distal regions of the A- and C-helices and also across the entire B-helix. This distribution of Φ-values is typical of a nucleation–condensation mechanism, with the A- and C-helices coming together to form a nucleus around which the rest of the structure subsequently forms. There are a number of positions at which no Φ-value can be accurately determined because the ΔΔ*G*_D–N_ is too small for an accurate calculation of Φ. At the majority of these positions, the qualitative Φ-value (“low” or “high”), determined by examination of relevant chevron plots, agrees with the overall pattern of Φ-values.

The unfolding Φ_u_^6M^-values cannot be compared with one another in the same detailed way, as it is not at all clear that they are reporting on the same rate-limiting transition state. However, as the majority of the Φ_u_^6M^-values are greater than the Φ_f_-values, they do indicate a growth of structure as the domain crosses the transition-state barrier(s).

### Comparing R15 with R16 and R17

How does this early transition state then compare with those of R16 and R17? Φ-Value analyses of R16 and R17 have been previously completed in this laboratory.^13,15^ The R16 Φ-value analysis is extensive, and Φ-values are available for both the early and the late transition states. The Φ-value analysis of R17 is less complete in terms of the number of Φ-values calculated, and only the early transition state is experimentally accessible. However, molecular dynamics simulations of R17 agree with the experimentally determined Φ-values and give access to the late transition state. These results show that R16 and R17 fold via very similar pathways.^13^

The coverages of the Φ-value analyses of R15 and R16 are comparable, so the rate-limiting transition states in low denaturant conditions can be compared in detail. For the two domains, the overall pattern of Φ-values is the same (Fig. 4). The A- and C-helices have high Φ-values and show both secondary and tertiary contacts being formed, whereas the B-helix is unstructured in both transition states. There are, however, differences in the structural detail of the transition states of R15 and R16. In R16, a clear difference can be seen between the magnitude of the Ala-Gly scanning Φ-values and those of the core Φ-values in the C-helix and, to some extent, in the A-helix. Φ-Values that report on secondary structure formation are higher than those that report on tertiary structure formation. Furthermore, in R16, the A- and C-helices have significant secondary structure formed along their whole length, and tertiary interactions, although less well formed, are also present. This pattern of high secondary structure Φ-values and lower tertiary structure Φ-values places the folding mechanism of R16 somewhere towards a framework (diffusion–collision-like) mechanism,^3^ and R17 shows the same behaviour. R15, in contrast, clearly folds via a nucleation–condensation mechanism, still centred round the A- and C-helices, but only the centres of the helices interact, not the entire lengths.

Is this difference in the detail of the transition-state structure sufficient to cause R15 to fold (and, equally importantly, unfold) 3 orders of magnitude faster than R16 and R17? Although subtle differences are seen between the transition-state structure of R15, when compared with R16 and R17, the Φ-value analyses indicate that the overall structures are similar. The β-Tanford value for the rate-limiting transition state for all three domains is 0.6, suggesting that all three transition-state structures are equally compact. Furthermore, the average Φ-value for the transition state is the same (0.26 in R15 from 33 Φ-values; 0.25 in R16 from 35 Φ-values), suggesting that the same amount of structure is present in the rate-limiting transition states in water for R15 and R16.

### Comparison of the folding of spectrin domains with other three-helix bundle families

The other three-helix bundle protein family that has been studied in such depth, the homeodomain family, differs from spectrin domains in many respects. One characteristic that they share is that all the domains in one family fold via a similar rate-limiting transition state, but the folding mechanism is not conserved.^3–9^ In one member of the homeodomain family En-HD, the presence of residual helical structure leads to helices forming early without the need for stabilisation from tertiary contacts.^4–8^ This allows the folding to proceed rapidly via a classical diffusion–collision mechanism. Slower-folding members of the family, without intrinsically stable helices, must depend upon a successful search for the folding nucleus of secondary and tertiary contacts.^3^ Thus, En-HD, with a very high helical propensity, folds fastest, with the rate-limiting step being the collision of preformed helices, while proteins with the lowest helical propensity (e.g., hTRF1) fold most slowly, apparently via a nucleation–condensation mechanism. The PSBD family, although less well studied, also appears to follow this pattern of behaviour.^10–12^

Unlike the homeodomains, the folding rate constants of R15, R16, and R17 do not reflect helical propensity: R15, the fastest folder, has the lowest helical propensity (Fig. 3). Both R16 and R17 have the highest helical propensity in the C-helix and low helical propensity in the A-helix (in contrast to R15). An obvious possibility is that the differences in the pattern and/or magnitude of helical propensity result in the slow folding of R16 and R17. However, altering the helical propensity, as predicted by AGADIR,^34–37^ in R16 to resemble that seen in R15 (R16rh and R16ih) does not make R16 fold faster (Fig. 3). Helical propensity, an important determinant in the rate of folding of the homeodomain proteins, therefore seems to be unimportant in determining the folding rate of the spectrin domains. (We note, however, that none of the spectrin domains has a helical propensity close to the ∼ 70% score found in En-HD. The difference in the helical propensities in R16rh and R16ih compared with that in wild-type R16 is significantly less than that seen between the fast-folding En-HD and the c-Myb-transforming protein, which sits somewhere along the continuum from a framework to a nucleation–condensation mechanism^3^ and may be too subtle to be picked up from folding rates.)

### Can we explain slow folding in R16 and R17?

Both folding and unfolding are faster in R15 than in R16 (and R17), so we chose to look at differences in transition-state structures. Although the stability of the two transition states cannot be directly measured, if the differences in the folding and unfolding rate constants of R15 and R16 were entirely due to differences in transition-state structure (with the transition state of R15 being significantly more stable—relative to both the denatured state and the native state—than that of R16), we would have predicted that the transition state of R15 should be significantly more structured than that of R16. This is not what we observe. We do, however, see that R15 folds by a nucleation–condensation mechanism, in contrast to R16 (and R17).

The early transition state of R15 has less extensive helical structure than those of R16 and R17. The B-helix of the spectrin domains consists of nine turns of helix, hence its long rod-like shape (Fig. 4). By comparison, the homeodomain family, the PSBD family, and the B domain of protein A all have four or fewer turns of helix in the longest helix.^11,40–43^ Setting up the correct register for these long helices in the spectrin domains (and, thus, native-state topology) must be a critical step in the folding pathway. The transition state of R15 shows how the fast-folding R15 achieves this. By the early transition state, the centres of the A- and C-helices have come together, with strong tertiary contacts holding the nascent helices in the correct register, supported by a couple of turns of helix. Once this correct long-range interaction has been set up, the rest of the structure can rapidly condense around the nucleus in a classical nucleation–condensation mechanism. This is not the case for the slower-folding R16 and R17. The early transition states of R16 and R17 lack this strong tertiary interaction between the A- and C-helices. The strongest interactions seen are secondary structure contacts formed along the entire length of the A- and C-helices. Some tertiary contacts are seen between the two helices; however, they are weaker and more diffuse than those seen for R15. For this mechanism, sitting on the continuum towards a more diffusion–collision mechanism, we might envisage that this lack of strong contacts between the long A- and C-helices leads to a more protracted search for the native-state topology. Many small-scale diffusion events between the A-helices and the C-helices could occur before a collision sets up the correct native-state topology and the folding becomes productive. A search such as this for the correct packing of the A- and C-helices would cause R16 and R17 to fold and unfold slower than expected, as is indicated by their status as slow-folding outliers on both the RCO and the LRO *versus* log*k*_f_ plots.

We have recently engineered fast-folding variants of R16 and R17 by making a number of mutations in each so that this hypothesis can thus be tested.

## Summary

The Φ-value analysis of R15 does not answer the question as to why it folds and unfolds so much faster than its homologues R16 and R17. There are indications that R15 folds along a similar pathway to R16 and R17 via a high-energy intermediate. The rate-limiting transition state of R15 has been characterised and compared with those for R16 and R17. The overall transition-state structure is similar, although subtle differences indicate that, as in the homeodomain family and the PSBD family, the folding mechanism is not conserved. We infer that the huge differences in folding and unfolding rate constants cannot be explained with reference to differences in transition-state structure. It is possible that the solution lies in the differences in folding mechanism. We note that a critical rate-determining event is the establishment of the correct topology so that long-range interactions, which are key for correct packing of the A- and C-helices in these large spectrin domains, can be established.

## Materials and Methods

### Protein expression and purification

Wild-type R15, R16, and R17 were produced as described.^16^ Site-directed mutagenesis of both R15 and R16 was performed using a Quikchange kit from Stratagene, and the identity of the mutants was confirmed by DNA sequencing.

Protein expression was carried out in *Escherichia coli* C41(DE3)^44^ grown in 2× TY media at 37 °C. Expression was induced once the cells reached an optical density at 600 nm of 0.4–0.6 AU by adding IPTG to a final concentration of 100 μg ml^− 1^ and by reducing the temperature to 26 °C. The cells were grown overnight and harvested by centrifugation. The harvested cells were sonicated and centrifuged, and the protein was purified from the soluble fraction by affinity chromatography on Ni^2+^-agarose resin. Bound protein was removed by thrombin cleavage and further purified by gel filtration. All proteins were stored at 4 °C.

### Stability

The stability of wild-type and mutant proteins was determined by urea-induced equilibrium denaturation, followed by changes in intrinsic (Trp) fluorescence on a Perkin-Elmer LS-55 luminescence spectrophotometer or by changes in ellipticity at 222 nm on an Applied Photophysics Chirascan circular dichroism spectrometer. All experiments used 1 μM protein in 50 mM sodium phosphate buffer (pH 7.0) and were carried out at either 10 °C or 25 °C.

### Kinetics

Folding and unfolding were monitored by following the change in intrinsic fluorescence on an SX18 or an SX20 stopped-flow spectrometer (Applied Photophysics) maintained at 10± 0.1 °C or 25 ± 0.1 °C. For experiments carried out at 10 °C, all solutions were chilled in the machine for 2 min before use. The final protein concentration used was 1 μM in 50 mM sodium phosphate buffer (pH 7.0). At least six traces were averaged, and the data were fitted to either a single-exponential process (R15, R17, and the unfolding arm of R16) or a double-exponential process (the refolding arm of R16). Due to mixing artifacts, data collected in the first 2.5 ms were always removed before fitting.

## Figures and Tables

**Table 1:**
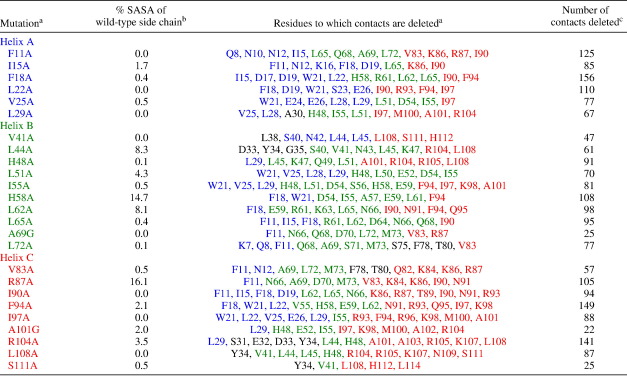
Contacts deleted upon mutation of core residues

^a^Residues from helix A are shown in blue, those from helix B are shown in green, and those from helix C are shown in red. Loop residues are shown in black. ^b^The percentage of solvent-exposed surface area (SASA) *versus* total surface area for the side chain of each core residue. ^c^The number of heavy-atom side-chain contacts within 6 Å of the deleted side chain. Data were calculated using the R15 structure in PDB file 1u4q^18^ using the program InsightII (Accelrys, Inc.).

**Table 2:** Kinetic and thermodynamic parameters for core mutants

Mutant	Δ*G*_D–N_^H_2_O^ (kcal mol^− 1^)^a^	ΔΔ*G*_D–N_^H_2_O^ (kcal mol^− 1^)	Δ*G*_kin_^H_2_O^ (kcal mol^− 1^)	*k*_f_^H_2_O^ (s^− 1^)^b^	*m*_*k*___u__ (M^− 1^)	*k*_u_^6M^ (s^− 1^)	Φ_f_^c^^,^^d^	Φ_u_^6M^^c,^^d^
Wild type	8.23 ± (0.05)	—	7.2 ± (0.2)	13,110 ± (100)	1.18 ± (0.01)	41 ± (3)	—	—
Helix A								
F11A	6.55 ± (0.04)	1.68 ± (0.05)	5.2 ± (0.1)	11,100 ± (40)	0.83 ± (0.01)	154 ± (3)	0.06	0.56
I15A	7.63 ± (0.04)	0.60 ± (0.05)	6.8 ± (0.2)	9760 ± (80)	1.26 ± (0.01)	107 ± (6)	—	—
F18A	4.08 ± (0.03)	4.15 ± (0.03)	3.5 ± (0.1)	2110 ± (20)	0.97 ± (0.01)	1486 ± (64)	0.25	0.51
L22A	6.30 ± (0.04)	1.93 ± (0.04)	5.8 ± (0.1)	1950 ± (20)	1.23 ± (0.01)	98 ± (5)	0.55	0.74
V25A	6.49 ± (0.04)	1.74 ± (0.04)	5.8 ± (0.1)	3690 ± (30)	1.23 ± (0.01)	205 ± (8)	0.41	0.48
L29A	5.91 ± (0.04)	2.32 ± (0.04)	5.5 ± (0.1)	1400 ± (10)	1.26 ± (0.01)	149 ± (9)	0.54	0.68
Helix B								
V41A	7.83 ± (0.05)	0.40 ± (0.05)	7.3 ± (0.1)	10,050 ± (40)	1.34 ± (0.01)	68 ± (2)	—	—
L44A	7.63 ± (0.04)	0.60 ± (0.05)	6.9 ± (0.2)	8730 ± (70)	1.23 ± (0.01)	62 ± (4)	—	—
H48A	6.87 ± (0.04)	1.36 ± (0.05)	6.1 ± (0.3)	17,720 ± (170)	1.10 ± (0.01)	247 ± (13)	—	—
L51A	5.36 ± (0.03)	2.87 ± (0.04)	4.4 ± (0.1)	3270 ± (10)	1.02 ± (0.01)	561 ± (18)	0.27	0.49
I55A	2.48 ± (0.02)	5.75 ± (0.03)	2.6 ± (0.1)	1510 ± (10)	1.03 ± (0.01)	7092 ± (413)	0.21	0.49
H58A	7.82 ± (0.04)	0.41 ± (0.05)	6.8 ± (0.3)	26,010 ± (200)	1.06 ± (0.01)	92 ± (5)	—	—
L62A	5.49 ± (0.03)	2.74 ± (0.04)	4.7 ± (0.1)	4710 ± (30)	1.05 ± (0.01)	553 ± (26)	0.21	0.46
L65A	5.49 ± (0.04)	2.74 ± (0.04)	4.0 ± (0.2)	7120 ± (60)	0.72 ± (0.01)	432 ± (12)	0.13	0.51
A69G	6.75 ± (0.04)	1.48 ± (0.05)	5.7 ± (0.1)	11,870 ± (60)	0.95 ± (0.01)	143 ± (4)	0.04	0.52
L72A	6.68 ± (0.04)	1.55 ± (0.05)	5.9 ± (0.2)	12,640 ± (120)	0.97 ± (0.01)	123 ± (6)	0.01	0.60
Helix C								
V83A	7.07 ± (0.04)	1.16 ± (0.05)	5.2 ± (0.1)	7510 ± (30)	0.72 ± (0.01)	56 ± (2)	0.27	0.85
R87A	7.65 ± (0.05)	0.58 ± (0.05)	6.9 ± (0.1)	10,930 ± (50)	1.19 ± (0.01)	58 ± (3)	—	—
I90A	6.90 ± (0.04)	1.33 ± (0.05)	6.5 ± (0.2)	9180 ± (90)	1.25 ± (0.01)	151 ± (9)	0.15	0.44
F94A	3.90 ± (0.02)	4.33 ± (0.03)	4.1 ± (0.1)	1320 ± (10)	1.40 ± (0.01)	3896 ± (252)	0.30	0.41
I97A	6.47 ± (0.04)	1.76 ± (0.04)	6.7 ± (0.1)	2240 ± (20)	1.40 ± (0.01)	60 ± (4)	0.56	0.87
A101G	4.39 ± (0.03)	3.84 ± (0.04)	4.4 ± (0.1)	550 ± (10)	1.46 ± (0.01)	1344 ± (119)	0.46	0.49
R104A	7.10 ± (0.04)	1.13 ± (0.04)	6.0 ± (0.2)	13,850 ± (130)	0.99 ± (0.01)	115 ± (5)	− 0.03	0.48
L108A	5.96 ± (0.04)	2.27 ± (0.04)	5.2 ± (0.2)	9450 ± (70)	1.10 ± (0.01)	607 ± (27)	0.08	0.33
S111A	8.13 ± (0.05)	0.10 ± (0.05)	7.8 ± (0.1)	14,250 ± (80)	1.30 ± (0.01)	33 ± (2)	—	—

a The value given is taken from equilibrium denaturation experiments. Δ*G*_D–N_^H_2_O^ is calculated from Δ*G*_D–N_^H_2_O^ = *m*_D–N_[urea]_50%_ using a mean *m*_D–N_ value of 1.88 kcal mol^−^ ^1^ M^−^ ^1^.

b Chevrons fitted globally with a shared *m*_*k*___f__ of 1.84 M^−^ ^1^.

c Φ-Values are only calculated for mutations where ΔΔ*G*_D–N_^H_2_O^ ≥ 0.75 kcal mol^−^ ^1^ (see the text and Fig. S3).

d Errors in Φ-values were propagated from errors of the fits of the chevron plots and ΔΔ*G*_D–N_ and are ≤ 0.05 (mean error: 0.01).

**Table 3:** Kinetic and thermodynamic parameters for Ala-Gly scanning mutants

Mutant	Δ*G*_D–N_^H_2_O^ (kcal mol^−^ ^1^)^a^	ΔΔ*G*_D–N_^H_2_O^ (kcal mol^−^ ^1^)^b^	Δ*G*_kin_^H_2_O^ (kcal mol^−^ ^1^)	*k*_f_^H_2_O^ (s^−^ ^1^)^c^	*m*_*k*___u__ (M^−^ ^1^)	*k*_u_^6M^ (s^−^ ^1^)	Φ_f_^d^^,^^e^	Φ_u_^6M^^d,^^e^
Wild type	8.23 ± (0.05)	—	7.2 ± (0.2)	13,110 ± (100)	1.18 ± (0.01)	41 ± (3)	—	—
Helix A								
Q9A	7.97 ± (0.05)	—	6.8 ± (0.1)	9040 ± (60)	1.06 ± (0.01)	28 ± (2)	—	—
Q9G	7.52 ± (0.04)	0.44 ± (0.06)	5.7 ± (0.2)	8060 ± (70)	0.79 ± (0.01)	37 ± (3)	—	—
T13A	8.38 ± (0.05)	—	8.0 ± (0.1)	13,270 ± (60)	1.34 ± (0.01)	27 ± (1)	—	—
T13G	7.44 ± (0.04)	0.94 ± (0.07)	6.7 ± (0.1)	11,530 ± (40)	1.16 ± (0.01)	90 ± (2)	0.08	0.29
K16A	8.84 ± (0.05)	—	8.0 ± (0.1)	17,620 ± (40)	1.28 ± (0.01)	25 ± (1)	—	—
K16G	7.54 ± (0.04)	1.30 ± (0.07)	6.7 ± (0.1)	13,800 ± (70)	1.14 ± (0.01)	87 ± (3)	0.11	0.46
F20A	8.48 ± (0.05)	—	8.0 ± (0.2)	29,560 ± (170)	1.27 ± (0.01)	39 ± (2)	—	—
F20G	7.94 ± (0.05)	0.54 ± (0.07)	7.0 ± (0.1)	11,370 ± (70)	1.23 ± (0.01)	68 ± (3)	—	—
E24A	7.91 ± (0.04)	—	7.6 ± (0.1)	9670 ± (60)	1.32 ± (0.01)	38 ± (2)	—	—
E24G	7.63 ± (0.04)	0.28 ± (0.06)	7.1 ± (0.1)	3080 ± (20)	1.29 ± (0.01)	25 ± (2)	—	—
E26A	6.35 ± (0.04)	—	6.1 ± (0.1)	2430 ± (20)	1.34 ± (0.01)	137 ± (6)	—	—
E26G	5.60 ± (0.04)	0.75 ± (0.05)	5.6 ± (0.1)	870 ± (10)	1.37 ± (0.01)	161 ± (7)	0.77	0.88
A27G	7.56 ± (0.04)	0.67 ± (0.06)	7.0 ± (0.1)	3520 ± (30)	1.33 ± (0.01)	42 ± (3)	—	—
Helix B								
A39G	7.72 ± (0.05)	0.51 ± (0.07)	6.9 ± (0.1)	12,760 ± (60)	1.18 ± (0.01)	73 ± (3)	—	—
N43A	8.52 ± (0.05)	—	7.8 ± (0.1)	17,350 ± (80)	1.24 ± (0.01)	28 ± (1)	—	—
N43G	7.56 ± (0.04)	0.96 ± (0.07)	6.9 ± (0.1)	11,950 ± (50)	1.27 ± (0.01)	108 ± (3)	0.22	0.21
K46A	8.16 ± (0.05)	—	5.8 ± (0.1)	9360 ± (60)	0.73 ± (0.01)	23 ± (2)	—	—
K46G	7.21 ± (0.04)	0.96 ± (0.06)	5.5 ± (0.1)	7090 ± (40)	0.89 ± (0.01)	80 ± (4)	0.16	0.26
L50A	7.69 ± (0.04)	—	7.4 ± (0.1)	8770 ± (40)	1.27 ± (0.01)	35 ± (1)	—	—
L50G	6.50 ± (0.04)	1.19 ± (0.06)	6.3 ± (0.1)	5920 ± (50)	1.27 ± (0.01)	162 ± (9)	0.19	0.28
A53G	6.96 ± (0.04)	1.28 ± (0.06)	6.3 ± (0.1)	7670 ± (60)	1.21 ± (0.01)	144 ± (6)	0.24	0.44
A57G	6.89 ± (0.04)	1.35 ± (0.06)	5.5 ± (0.1)	3600 ± (20)	0.93 ± (0.01)	57 ± (3)	0.54	0.86
D60A	8.30 ± (0.05)	—	7.5 ± (0.1)	15,880 ± (40)	1.25 ± (0.01)	49 ± (1)	—	—
D60G	7.78 ± (0.05)	0.53 ± (0.07)	7.2 ± (0.1)	8880 ± (50)	1.28 ± (0.01)	53 ± (3)	—	—
K63A	7.89 ± (0.05)	—	7.5 ± (0.2)	11,490 ± (80)	1.29 ± (0.01)	43 ± (3)	—	—
K63G	7.07 ± (0.04)	0.82 ± (0.06)	6.4 ± (0.1)	7710 ± (40)	1.20 ± (0.01)	129 ± (7)	0.27	0.24
S67A	8.60 ± (0.05)	—	8.2 ± (0.1)	13,340 ± (50)	1.41 ± (0.01)	30 ± (1)	—	—
S67G	7.43 ± (0.04)	1.17 ± (0.07)	6.8 ± (0.2)	10,760 ± (100)	1.21 ± (0.01)	91 ± (6)	0.10	0.46
S71A	8.08 ± (0.04)	—	8.4 ± (0.1)	14,330 ± (50)	1.49 ± (0.01)	35 ± (2)	—	—
S71G	7.44 ± (0.04)	0.64 ± (0.06)	7.4 ± (0.1)	14,770 ± (70)	1.32 ± (0.01)	76 ± (3)	—	—
Helix C								
S81A	8.01 ± (0.05)	—	7.6 ± (0.1)	12,580 ± (60)	1.27 ± (0.01)	39 ± (2)	—	—
S81G	7.84 ± (0.05)	0.17 ± (0.07)	7.1 ± (0.1)	12,980 ± (70)	1.21 ± (0.01)	54 ± (2)	—	—
D85A	8.21 ± (0.05)	—	8.4 ± (0.2)	13,480 ± (110)	1.46 ± (0.01)	28 ± (3)	—	—
D85G	7.25 ± (0.04)	0.96 ± (0.06)	6.9 ± (0.2)	12,980 ± (80)	1.18 ± (0.01)	67 ± (4)	0.02	0.49
E88A	7.96 ± (0.05)	—	5.9 ± (0.1)	6510 ± (30)	0.77 ± (0.01)	19 ± (1)	—	—
E88G	7.21 ± (0.04)	0.75 ± (0.06)	5.3 ± (0.1)	5440 ± (30)	0.78 ± (0.01)	49 ± (2)	0.13	0.29
G92A	8.73 ± (0.05)	0.57 ± (0.07)	8.2 ± (0.3)	32,180 ± (210)	1.19 ± (0.01)	19 ± (2)	—	—
Q95A	7.77 ± (0.04)	—	7.6 ± (0.1)	12,990 ± (70)	1.30 ± (0.01)	44 ± (3)	—	—
Q95G	7.38 ± (0.04)	0.39 ± (0.06)	6.7 ± (0.1)	4990 ± (40)	1.28 ± (0.01)	68 ± (4)	—	—
S99A	8.44 ± (0.05)	—	7.7 ± (0.3)	26,620 ± (180)	1.21 ± (0.01)	43 ± (3)	—	—
S99G	7.46 ± (0.04)	0.98 ± (0.06)	7.4 ± (0.1)	7150 ± (50)	1.39 ± (0.01)	60 ± (3)	0.76	0.80
A103G	6.85 ± (0.04)	1.38 ± (0.06)	6.4 ± (0.1)	5420 ± (30)	1.32 ± (0.01)	157 ± (6)	0.36	0.45
A106G	7.08 ± (0.04)	1.16 ± (0.06)	6.6 ± (0.1)	7850 ± (30)	1.33 ± (0.01)	177 ± (4)	0.25	0.28
L114A	7.99 ± (0.05)	—	7.7 ± (0.2)	13,560 ± (90)	1.32 ± (0.01)	46 ± (3)	—	—
L114G	7.67 ± (0.04)	0.32 ± (0.06)	7.4 ± (0.1)	12,460 ± (50)	1.25 ± (0.01)	44 ± (2)	—	—

a The value given is taken from equilibrium denaturation experiments. Δ*G*_D–N_^H_2_O^ is calculated from Δ*G*_D–N_^H_2_O^ = *m*_D–N_[urea]_50%_ using a mean *m*_D–N_ value of 1.88 kcal mol^−^ ^1^ M^−^ ^1^.

b For Ala-Gly helix scanning positions, values ofΔΔ*G*_D–N_^H_2_O^, Φ_f_, and Φ_u_ are shown against the appropriate glycine mutant, except for G92A, where values are shown against the alanine mutation.

c Chevrons fitted globally with a shared *m*_*k*___f__ of 1.84 M^−^ ^1^.

d Φ-Values are only calculated for mutations where ΔΔ*G*_D–N_^H_2_O ^≥ 0.75 kcal mol^−^ ^1^ (see the text and Fig. S3).

e Errors in Φ-values were propagated from errors of the fits of the chevron plots and ΔΔ*G*_D–N_ and are ≤ 0.05 (mean error: 0.01).
