# Supplementary material for: Different Members of a Simple Three-Helix Bundle Protein Family Have Very Different Folding Rate Constants and Fold by Different Mechanisms
Source: J Mol Biol. 2009 Jul 31;390(5):1074–85. doi: 10.1016/j.jmb.2009.05.010 (PMC2852649; doi:10.1016/j.jmb.2009.05.010)
Supplement: Supplementary material [file mmc1.pdf]

Supplementary Material for:

Different members of a simple 3-helix bundle protein family have very different folding rates and fold by different mechanisms.

Beth G. Wensley, Martina Gärtner, WanXian Choo, Sarah Batey, Jane Clarke.

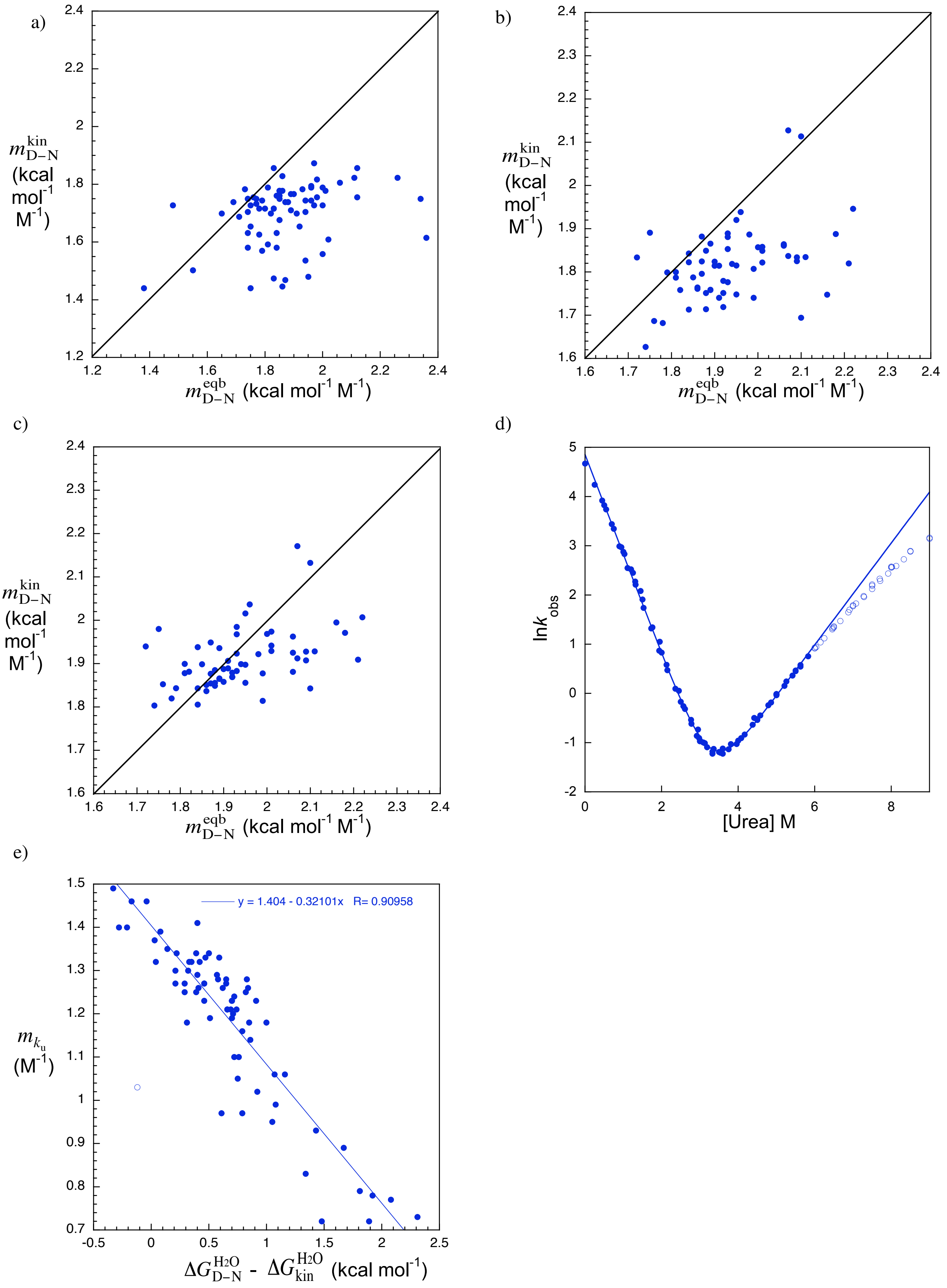

Supplementary Figure S1. The variation seen in m_{k_u} and its effects. (a) – (c) Comparisons of equilibrium (m^{eqb}_{D-N}) and kinetic (m^{kin}_{D-N}) m_{D-N} for (a) wild-type R15 and all mutants, (b) wild-type R16 and all mutants using only data collected between 0 and 6 M urea and fit with a

standard two-state chevron fit and (c) R16 and all mutants using all data collected and fit with a sequential transition state fit[1,2]. Lines have the equation $y = x$. (d) The chevron plot for wild-type R16 at 25 ˚C. Data collected > 6 M urea have open circles and were not included in the fit. The continuous line represents the fit of the filled circles to a standard two-state chevron fit with parameters: $k_f^{H_2O}$ = 128 s^{-1}, $k_u^{H_2O}$ = 5 x 10^{-3} s^{-1}, m_{k_f} = 2.0 M^{-1}, m_{k_u} = 1.0 M^{-1}. (e) Effect of m_{k_u} on $\Delta G_{D-N}^{H_2O}$ - $\Delta G_{kin}^{H_2O}$ for wild-type R15 and all mutants. The equation shows a linear fit to the data. The open circle is I55A, which has a very short unfolding arm due to a low stability and low Φ-value, and has been excluded from the fit.

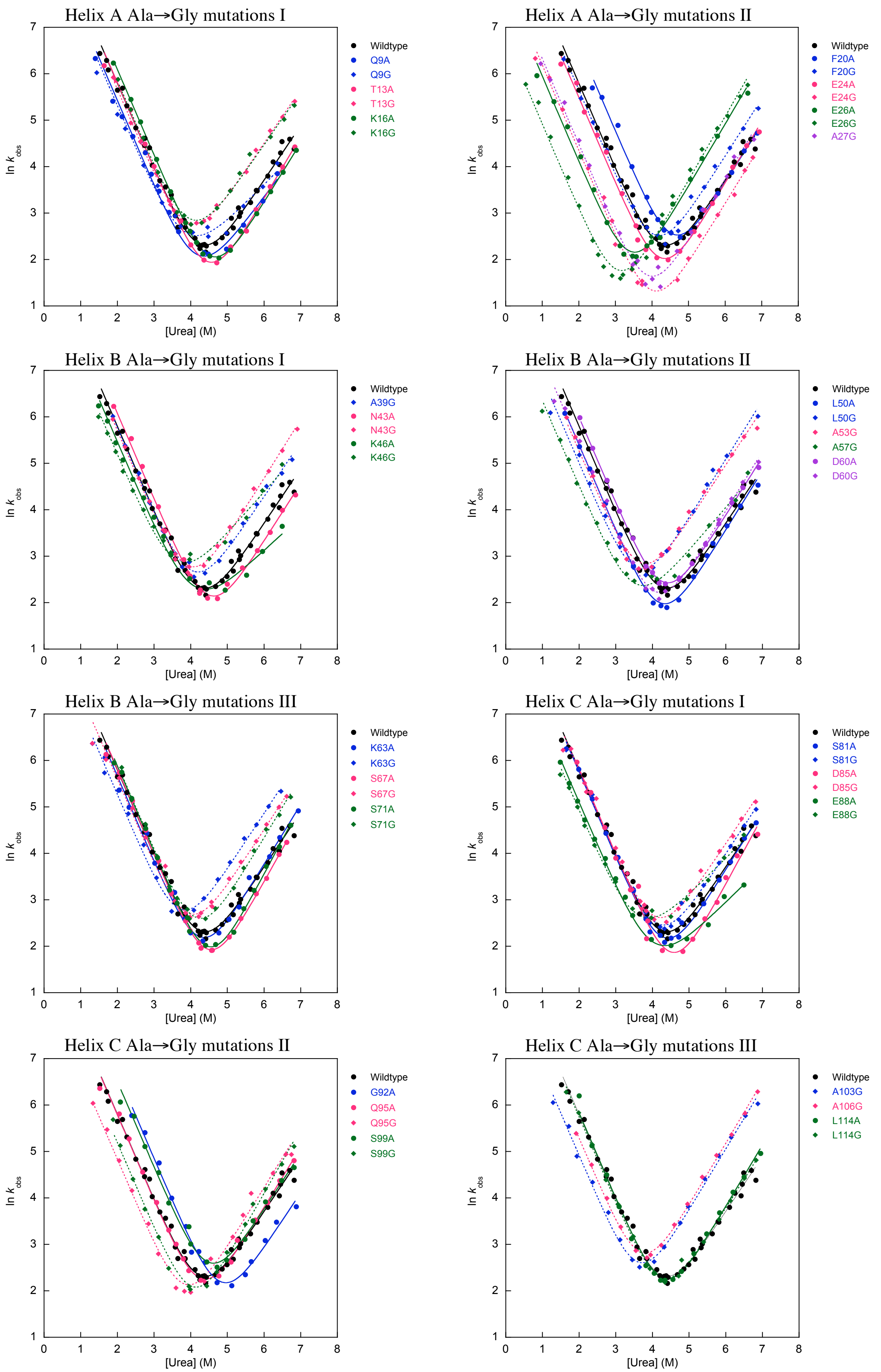

Supplementary Figure S2

Supplementary Figure S2. Chevron plots and fits for R15 Ala-Gly scanning mutations. Continuous lines represent the fit for each mutant to a globally fitted two-state fit with a shared m_{k_f}.

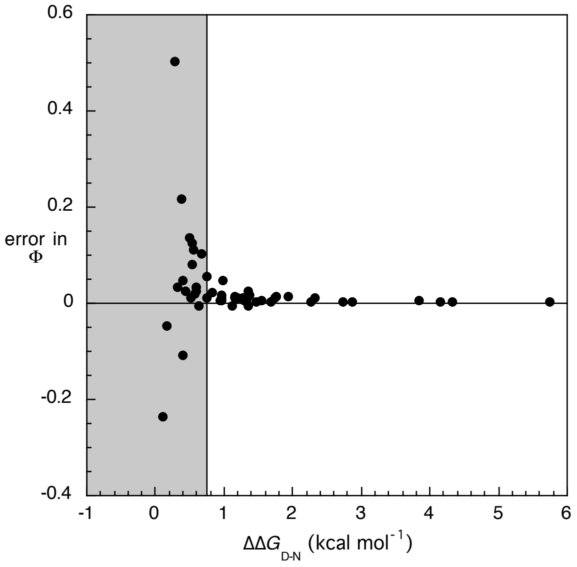

Supplementary Figure S3. The errors in Φ are low where $\Delta\Delta G_{D\text{-}N} \leq 0.75$. This was the cut-off used in our study.

References

1. Scott, K. A. & Clarke, J. (2005). Spectrin R16: broad energy barrier or sequential transition states? *Protein Sci.* **14**, 1617-1629.

2. Scott, K. A., Randles, L. G. & Clarke, J. (2004). The folding of spectrin domains II: phi-value analysis of R16. *J. Mol. Biol.* **344**, 207-221.
